# Diamond Coating Reduces Nuclear Fuel Rod Corrosion at Accidental Temperatures: The Role of Surface Electrochemistry and Semiconductivity

**DOI:** 10.3390/ma14216315

**Published:** 2021-10-22

**Authors:** Lucie Celbová, Petr Ashcheulov, Ladislav Klimša, Jaromír Kopeček, Kateřina Aubrechtová Dragounová, Jakub Luštinec, Jan Macák, Radek Škoda, Irena Kratochvílová

**Affiliations:** 1 Institute of Physics of the Czech Academy of Sciences, Na Slovance 1999/2, 182 21 Prague, Czech Republic; celbova@fzu.cz (L.C.); ashcheulov@fzu.cz (P.A.); klimsa@fzu.cz (L.K.); kopecek@fzu.cz (J.K.); dragounova@fzu.cz (K.A.D.); lustinec@fzu.cz (J.L.); 2 Faculty of Nuclear Sciences and Physical Engineering, Czech Technical University in Prague, Břehová 7, 115 19 Prague, Czech Republic; 3 Power Engineering Department, University of Chemistry and Technology, Technická 3, 166 28 Prague, Czech Republic; Jan.Macak@vscht.cz; 4 Czech Institute of Informatics, Robotics and Cybernetics, Czech Technical University in Prague, Jugoslávských partyzánů 1580/3, 160 00 Prague, Czech Republic; Radek.Skoda@cvut.cz

**Keywords:** diamond coating, ZIRLO, surface electrochemistry, chemical vapor deposition, nuclear fuel rods corrosion

## Abstract

If we want to decrease the probability of accidents in nuclear reactors, we must control the surface corrosion of the fuel rods. In this work we used a diamond coating containing <60% diamond and >40% sp^2^ “soft” carbon phase to protect Zr alloy fuel rods (ZIRLO^®^) against corrosion in steam at temperatures from 850 °C to 1000 °C. A diamond coating was grown in a pulse microwave plasma chemical vapor deposition apparatus and made a strong barrier against hydrogen uptake into ZIRLO^®^ (ZIRLO) under all tested conditions. The coating also reduced ZIRLO corrosion in hot steam at 850 °C (for 60 min) and at 900 °C (for 30 min). However, the protective ability of the diamond coating decreased after 20 min in 1000 °C hot steam. The main goal of this work was to explain how diamond and sp^2^ “soft” carbon affect the ZIRLO fuel rod surface electrochemistry and semi conductivity and how these parameters influence the hot steam ZIRLO corrosion process. To achieve this goal, theoretical and experimental methods (scanning electron microscopy, Raman spectroscopy, electrochemical impedance spectroscopy, carrier gas hot extraction, oxidation kinetics, ab initio calculations) were applied. Deep understanding of ZIRLO surface processes and states enable us to reduce accidental temperature corrosion in nuclear reactors.

## 1. Introduction

Although the percentage of electricity produced worldwide via renewable energy resources is gradually increasing year over year, nuclear energy remains one of the most efficient and reliable sources of a continuous supply of electricity. Nevertheless, significant complications during an operation/exploitation of nuclear power plants are associated with corrosive processes in nuclear fuel rods [1,2,3,4]. Being in contact with the coolant, nuclear fuel rods are oxidized and absorb hydrogen that contributes to the embrittlement of nuclear fuel rods. In this way the operability and integrity of rods at regular (300–400 °C) and accidental temperature conditions (above 800 °C) are reduced. In Fukushima (2011), fuel rods corrosion occurred at accidental temperatures so high that the radioactive products leaked into the environment [2,5,6,7].

At present, zirconium-based alloys are essential materials used in the form of rods to prevent direct contact of the nuclear fuel with the environment of the reactor [8,9,10,11,12]. 

The commonly followed strategy to protect zirconium alloys against the above-described corrosion is to protect nuclear fuel rod surfaces by a water impermeable coating exhibiting lower oxidation potential than the zirconium alloy itself [2,13,14,15]. A disadvantage of such a strategy is that its effectiveness depends strongly on the structural integrity of the implemented coating [16,17]. Coating defects arise as a result of substrate volume changes with temperature.

In our recent works, we showed that a water-permeable polycrystalline diamond film (PCD) [18,19,20,21,22,23,24], which contained more than 95% of diamond and fully covered Zr alloy fuel rods, effectively reduced Zr-alloy nuclear fuel rod hot steam/water corrosion. 

In this work, using experimental approaches complimented with theoretical/computational modelling, we described and explained the influence of diamond coating on chemical and physical processes and states that affect the hot steam ZIRLO corrosion process. We focused on the contribution of the carbon phases [25] and steam temperature to the formation of surface complexes that affect Zr corrosion. Specially, we demonstrated the ability of a coating containing less than 60% diamond phase and >40% sp^2^ carbon phase to protect ZIRLO nuclear fuel rods against corrosion at nuclear reactor accidental temperatures (850 °C to 1000 °C). We showed how ZrO_2_ semi conductivity and surface chemical complexes affected the water dissociation processes and highlighted the importance of diamond coating composition (sp^3^ and sp^2^ carbon phases) in the protection of ZIRLO nuclear fuel rods against hot steam corrosion. The diamond coating was grown in a pulse microwave plasma chemical vapor deposition apparatus. Generally, the production of such coating places lower demands on the technological equipment and procedures compared to PCD (>95% diamond) coating technology.

## 2. Materials and Methods

### 2.1. Diamond Coating

Essentially, Zr-alloy rods (Sn 1–0.7%; Fe 0.1%; O 0.13%; Nb 1%—further referred in the text as ZIRLO) of 25 mm in length were cleaned and loaded into the pulsed microwave plasma enhanced chemical vapor deposition apparatus with linear antenna delivery [20,25]. An H_2_ + CH_4_ + CO_2_ gas mixture, a pressure of 0.3 mbar, a pulse microwave power of 2 × 3 kW, and a temperature of 600 °C were utilized for the growth of coatings on ZIRLO surfaces. To ensure the formation of coatings on the entire surface of ZIRLO rods, the whole outer circumference of rods was exposed to the plasma during the coating process. 

### 2.2. Scanning Electron Microscopy, Raman Spectroscopy

The surface morphologies of the diamond layers were analyzed using a Tescan FERA 3 scanning electron microscope (TESCAN, Brno, Czech Republic). To minimize the interaction volume during imaging, the accelerating voltage in high-resolution mode was kept in the range of 5 kV. The structures were opened using a Xenon plasma Focused-Ion Beam (Xe FIB). Conventional metallographic samples were also prepared for elemental mapping. 

The diamond coating composition (sp^2^ and sp^3^-hybridized carbon) was characterized with Raman spectroscopy performed at room temperature using a Renishaw InVia Reflex Raman microscope (488 nm (5 mW) (Renishaw plc, New Mills, Wotton–under–Edge, England, Great Britain), a 50× Olympus objective, 65 µm slits, a spot focus of ~1 µm, and a grating of 2400 lines/mm. Spectra were acquired at various points across the samples to probe the homogeneity of the deposited carbon coatings. The Raman maps were collected on Horiba LabRAM HR Evolution (HORIBA France SAS, Villeneuve d’Ascq, France) with a 532 nm excitation wavelength, laser power of 5 mW, and a 100× Olympus objective and ~1 µm spot focus. The maps were acquired with steps of 1 µm in both x and y directions.

### 2.3. Oxidation Kinetics

To simulate the ability of the diamond coating to protect ZIRLO cladding against oxidation at accident-specific hot steam conditions in a nuclear reactor, the coated ZIRLO fuel cladding samples were subjected to a 60 min 850 °C hot steam furnace test or to 30 min 900 °C and 20 min 1000 °C tests. A Netzsch STA449 F3 Jupiter (NETZSCH-Gerätebau GmbH, Selb, Germany) was used for the oxidation kinetics of all samples. A steam furnace was connected with an Aeolos mass spectrometer (NETZSCH-Gerätebau GmbH, Selb, Germany). Heating was performed in Ar at 20 K/min, and cooldown was performed in Ar at 100 K/min. Steam injection started 5 min after reaching the isothermal plateau until the end of the isothermal phase(s). The atmosphere during the oxidation phase was 2 g/h H_2_O + 50 mL/min Ar (protective gas), corresponding to P_H2O_ = 0.45 bar. The chamber pressure was approximately 1 bar. The comparable steam flow conditions were fixed in the experimental equipment.

The relative weight gain was obtained as the difference between the sample weight before and after oxidation. The weight of the material released during the hot steam/autoclave treatments was included. The weight gains of all the samples were normalized to the relevant/coated area of the rod surface; the inner surfaces of the ZIRLO rods were not fully covered by the carbon layer, as the coating was unable to fully penetrate into a rod’s internal structure. We recalculated the weight gain of the coated parts of the rods (fully covered the outer surface and covered 40% of the inner surface). From these results, weight gain of the coated parts of the samples in mg∙dm^−2^ was calculated.

### 2.4. Carrier Gas Hot Extraction

Absorbed hydrogen content in hot steam processed carbon coated and uncoated ZIRLO samples was estimated by carrier gas hot extraction (CGHE). CGHE measurements were carried out using a Bruker G8 Galileo ON/H Analyzer (Bruker AXS GmbH, Karlsruhe, Germany). The amount of hydrogen was measured by a thermal conductivity detector. The carrier gas was nitrogen. Hydrogen was extracted at melting temperatures by using an electrical furnace.

### 2.5. Electrochemical Impedance Spectroscopy

Characterization of the charge transfer properties of diamond layer-coated and uncoated samples before and after simulation of the accident-specific hot steam conditions in a nuclear reactor was performed using electrochemical impedance spectroscopy (EIS). EIS data were obtained at room temperature in a demineralized water solution of K_2_SO_4_ (0.5 M) in a 3-electrode cell. An Ag–AgCl (1 M) electrode was used as a reference electrode, and ZIRLO rod samples approximately 2.5 cm long were used as working electrodes. Part of the oxide layer on the internal surface was removed by a microcutter to establish electric contact, and the samples were placed in special holders. The edges and internal surfaces of the rods were isolated by silicon rubber sealing and (polytetrafluoroethylene) PTFE tape so that only the outer surfaces of the rods was exposed. The total exposed area of the samples was 6.6 cm^2^. A coaxial platinum mesh around the working electrode served as the counter electrode. A Reference 600+ potentiostat (Gamry) was used for the measurements. EIS measurements were performed in potentiostatic mode at the open-circuit potential (OCP). Usually, 1 h was necessary to stabilize the OCP. The frequency range was usually 1 × 10^6^−1 × 10^−2^ Hz. The amplitude of the perturbation signal was 5 mV. EIS spectra were analyzed using ZSimpWin 3.21 (Ametek), ZView 3.2 (Scribner Associates), and Origin 8.5 (OriginLab) software.

### 2.6. Ab Initio Calculations

Density functional theory (DFT) was used to calculate the absorption and dissociation energies of water molecules on the ZrO_2_ (−111) surface. The calculated system was composed of 4.795 Å thick ZrO_2_ and 10 Å wide vacuum. Furthermore, the ZrC (111) surface was simulated to investigate water dissociation upon the possible integration of carbon into ZIRLO. These calculations were performed using CASTEP code (full-featured materials modelling code based on a first-principles quantum mechanical description of electrons and nuclei) [26,27] in Materials Studio 8.0 with ultrafine settings of the ultrasoft pseudopotential and Perdew–Burke–Ernzerhof (PBE) exchange-correlation functional exchange and correlation functional to reduce the computational resources [28].

## 3. Results

### 3.1. Scanning Electron Microscopy, Raman Spectroscopy

**Figure 1:**
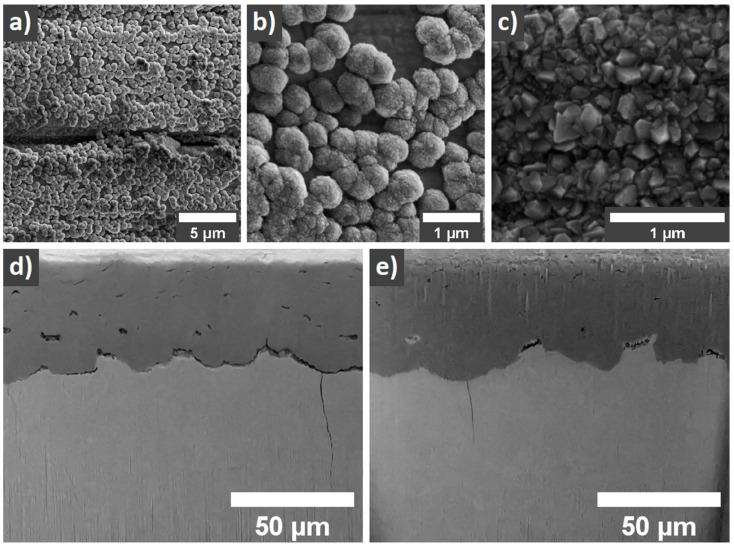
Scanning electron microscopy of as-prepared and hot steam-treated-coated ZIRLO fuel rods. (**a**,**b**) SEM of the carbon layer (<60% of diamond phase) coated ZIRLO samples as prepared. The low surface density coverage is clearly seen. (**c**) SEM of PCD coating (>95% of diamond phase) of ZIRLO samples as presented in our previous studies [1,2,3]. SEM images of ZIRLO samples after hot steam oxidation (30 min at 900 °C and 20 min at a 1000 °C in a hot steam furnace) showing structures unveiled using an Xe FIB (**d**,**e**). The sample without any protection (**d**) has an oxidation layer filled with massive cracks. The sample protected by the diamond coating (**e**) has a smaller number of cracks, both in the oxidized layer and at its boundary with ZIRLO. The oxidized layer can be distinguished as a darker area.

Following our recent reports [18,19,20], where a self-nucleation of diamond particles on Zr surfaces has been demonstrated, in this work we created a layer (diamond coating) 500 nm thick, where individual diamond (sp^3^) grains were surrounded by an outer shell comprised of sp^2^ bounded carbons (Figure 1a,b). As temperature presents a prime factor for safe and reliable operation, we studied the protective behavior and the properties of the diamond coatings during the simulation of the nuclear reactor in hot steam at accidental temperatures. The application of the hot steam for 60 min at 850 °C, 30 min at 900 °C, followed by 20 min at 1000 °C resulted in a significant change of the diamond coating as observed by SEM and Raman spectroscopy. Particularly, treatment of the ZIRLO rods with diamond coating and uncoated ZIRLO in the presence of the hot steam for 30 min at 900 °C and 20 min at 1000 °C resulted in the formation of a ca. 50 µm thick oxide-rich layer that expanded into the surface of the ZIRLO (Figure 1d,e, Figures S1 and S2 in Supplementary Material). The uncoated ZIRLO had an oxidation layer with massive cracks. The coated ZIRLO had a smaller number of cracks both in the oxidized layer and at its boundary with ZIRLO.

When comparing scanning electron microscopy (SEM) observations of diamond-coated ZIRLO samples to previously published PCD-coated ZIRLO rod (Figure 1c) surfaces, the herein fabricated layers displayed a low coverage density with areas where the coating is absent, hence exposing the bare surface of the ZIRLO rod (Figure 1b). The diamond coating covered less than 70% of the ZIRLO surface area.

**Figure 2:**
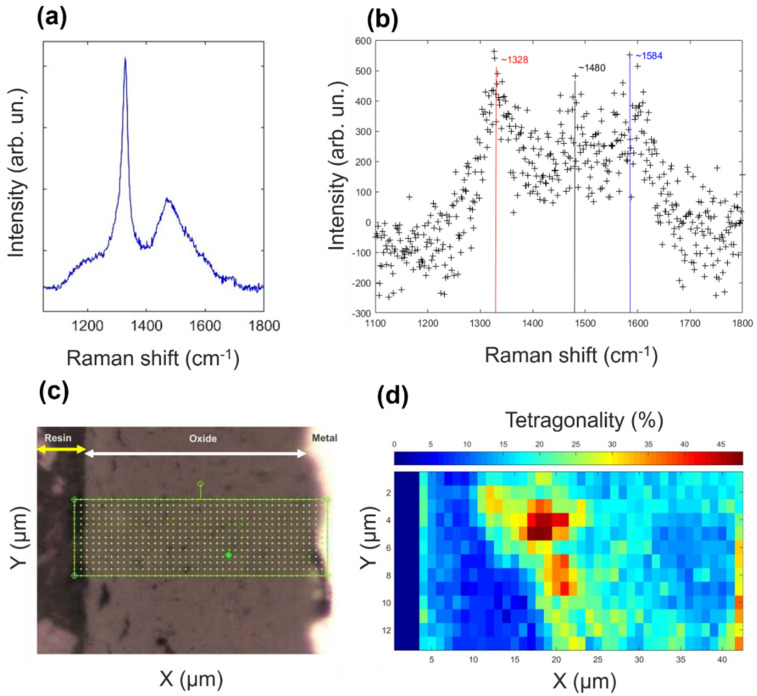
The as-prepared and high-temperature-treated ZIRLO rods characterized by non-destructive Raman spectroscopy. (**a**,**b**) Representative Raman spectra of the as-prepared carbon ZIRLO coating (coating contained less than 60% of diamond phase, spectra varied over the rod’s surface, see Figure S1 in Supplementary Material). (**b**) The diamond peak is seen at ~1328 cm^−^^1^ accompanied by a G peak at ~1580 cm^−^^1^, and a peak at ~1480 cm^−^^1^, which we assigned to amorphous carbon. (**c**) Optical image of the mapped region and (**d**) a map of percent tetragonality for oxidized coated ZIRLO. The first three columns were set to zero as they represent or are strongly affected by the surrounding epoxy resin.

As additionally supported by SEM characterizations (Figure 1a,b), Raman spectroscopy measurements showed that the as-prepared diamond coating consisted of diamond nano-grains/domains embedded in sp^2^ carbon, disordered graphite, and amorphous sp^2^ carbon. The peak associated with diamond exhibited a downshift from the typical position at ~1332 cm^−1^ to ca. 1328 cm^−1^ along with asymmetrical broadening to lower wavenumbers, assigned to a phonon confinement in the nano-sized diamond grains (Figure 2a,b). The type of the sp^2^ carbon phase in the coating was presented mainly by the stretching of sp^2^ carbons in the rings and chains (G band at ca 1580 cm^−1^) and by the “breathing” mode of sp^2^ carbons in rings (D band at ca. 1380 cm^−1^). Intensity and intensity ratio of those two bands varied across the rod surfaces (Figure S1). The peak at ~1534 cm^−1^ (Figure 2a) possibly belongs to amorphous carbon (a-C). The ~1480 cm^−1^ peak (Figure 2b) could be attributed to semicircle stretching of carbon atoms in aromatic rings [29] or to a-C.

The Raman spectra of the coating after hot steam treatment (see Figure S2 in Supplementary Material) exhibited a homogeneous distribution throughout the rod surface. Moreover, Raman spectra taken from the cross-section of the rod after the hot steam treatment suggest the presence of sp^2^ carbon up to the depth of ca. 7 µm into the rod’s surface. Additionally, calculation of the *m*/*I_G_* parameter (where m is the slope of the linear photoluminescence background, while *I_G_* is the intensity of the G band), which is used for the estimation of hydrogen content from Raman spectra (for example [30,31]), was 5–6 times higher in value for coated ZIRLO rods treated in the hot steam at 850 °C than after the 1000 °C steam treatment, as discussed in the Supplementary Material S1.

To explain the appearance of cracks in the oxide-rich layer of the ZIRLO rod surface after the hot steam exposure as observed by SEM (Figure 1c,d), the Raman spectra mapping of the oxide structure in the cross-section was performed, and the percent tetragonality was calculated following the approach presented in the work of Efaw et al. [32]. The mapping revealed the presence of tetragonal ZrO_2_ inside the bulk of the monoclinic ZrO_2_ (Figure 2d). The tetragonal phase could be associated with the reduced number of cracks seen in the case of coated ZIRLO rods in comparison with oxidized bare ZIRLO rods—lesser volume that passed through the change from the tetragonal to the monoclinic phase. Notably, from the performed spectral analysis, only the percent tetragonality with values higher than ~20% (Figure 2d) should be considered as a tetragonal-rich phase due to the inherent noise of the data. Additionally, the tetragonal-rich phase was not observed within the bulk of the monoclinic ZrO_2_ phase in the case of hot-steam-treated uncoated ZIRLO rods.

### 3.2. Oxidation Kinetics

As highlighted in previous paragraphs, exposure of the herein examined ZIRLO rods to hot steam at various temperatures allowed for the simulation of possible accidental conditions in the core of the nuclear reactor in the case of the loss of cooling water. Here, to mimic such conditions, the ZIRLO rod samples were exposed to the increasing temperature of the steam (850 °C for 60 min, 900 °C for 30 min, and 1000 °C for 20 min) and the oxidation kinetics was analyzed in terms of the oxidation weight increase.

At first, the exposure of the coated and uncoated ZIRLO samples to hot steam at 850 °C for 60 min resulted in the reduced oxidation (by 16%) of the ZIRLO rod protected by the diamond coating when compared to the uncoated ZIRLO rods. The weight gain values of hot steam-processed coated ZIRLO rod were 700 mg∙dm^−2^, while values of the hot steam-processed bare/uncoated ZIRLO rod samples were 790 mg∙dm^−2^.

**Figure 3:**
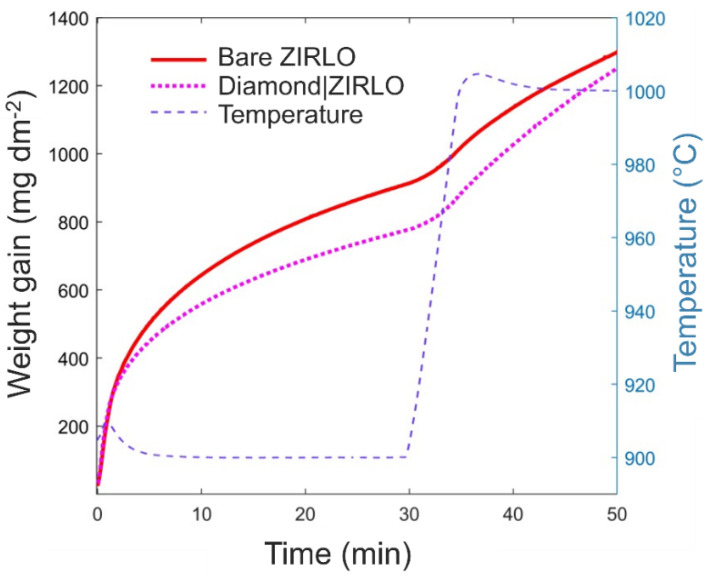
Oxidation kinetics—evolution of the weight gain during the hot steam treatment of bare and coated ZIRLO rods for 30 min at 900 °C followed by 20 min at ~1000 °C.

Following the treatment in the steam at 900 °C for 10 min, the oxidation of coated ZIRLO rods was reduced by 11% compared to that of uncoated ZIRLO rods treated under the same conditions. As the treatment time increased to 20 min in 900 °C, the oxidation of coated ZIRLO rods was further reduced by 20% compared to that of uncoated ZIRLO rods. Finally, after 30 min at 900 °C and then 20 min at 1000 °C in the hot steam, the oxidation of the coated ZIRLO rods was reduced by 7% compared to that of uncoated ZIRLO (see Figure 3 and the data in Table 1).

### 3.3. Effect of the Diamond Coating on the Hydrogen Uptake by ZIRLO Surfaces

Samples were also characterized for absorbed hydrogen by carrier gas hot extraction. The obtained results demonstrated that after performance of the hot steam treatment at 900–1000 °C, the uncoated ZIRLO rods exhibited a higher hydrogen concentration (1166 ppm) compared to the ZIRLO rod protected by the diamond coating (164 ppm). The ratio of the hydrogen content in the hot steam-processed-uncoated ZIRLO and ZIRLO rods with diamond coating corresponds well to our previous results—after hot steam (for 1100 °C and 1200 °C) the uptake of hydrogen into uncoated ZIRLO was an order of magnitude higher than in the case of the diamond layer protected ZIRLO rods [19].

### 3.4. Electrochemical Impedance Spectroscopy

**Figure 4:**
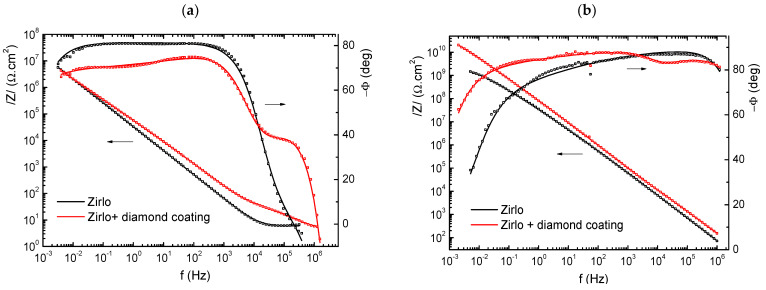
Bode plots of the impedance response of uncoated and diamond layer coated ZIRLO samples before (**a**) and after (**b**) exposure to steam at 850 °C.

Typical shapes of the impedance spectra are presented in Figure 4.

**Figure 5:**
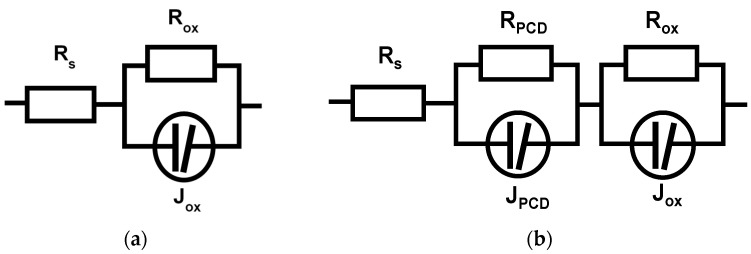
Equivalent circuits used for experimental impedance data fitting: (**a**) uncoated samples before and after exposure to 850 °C steam without diamond layer, and (**b**) diamond-coated samples. *R_s_*—electrolyte resistance, *R_ox_, J_ox_*—resistance and dispersive capacitance, respectively, forming an impedance loop at high frequencies; *R_lf_, J_lf_*—the same, but at lower frequencies.

Experimental data fitting was performed using the equivalent circuits shown in Figure 5.

The *J* elements describe the dispersive capacitance response formulated according to the universal law of the dielectric response suggested by Jonscher [33]. The impedance term of a J element can be expressed as

(1) $$Z_{J}=\left(Q^{-1}{\left(j\omega \right)}^{1-n}+C_{\infty }{}^{-1}\right){\left(j\omega \right)}^{-1}$$

where *C*_∝_ is defined as the “ideal” capacitance value at infinite frequency, *Q* and *n* are constants related to the dielectric dispersion, and *j* and *ω* have their usual meaning. From Equation (1), it is evident that the impedance of the dispersive capacitance has a formal form of a parallel connection of a constant phase element (CPE) and the “ideal” capacitance.

The bare ZIRLO sample had a relatively simple impedance spectrum. The sample was in a passive state, and the response came from very slow charge transfer across the native oxide (from the production phase of the rod) and the associated capacitance, which was rather low (approximately 1 μF·cm^−2^) and corresponded to a thin oxide layer or double layer at the oxide/electrolyte interface. The diamond coating significantly changed the impedance response. In the high frequency range (10^6^–10^3^ Hz), the highly dispersive impedance response (phase angle lower than 40°) of the diamond layer dominated. In addition, the overall impedance spectrum of the coated sample was more dispersed. This difference was evidently the effect of the diamond layer character and changed surface roughness.

The samples exposed to 850 °C showed the predominant oxide layer response over the whole range of frequencies. In this case, the level of oxide impedance dispersion at lower frequencies was higher than that of the uncoated samples. Nevertheless, quite interestingly, the response of the remnants of the diamond coating could still be seen in the shape of the phase angle frequency dependence in approximately the same frequency range as the original PCD coating (10^6^–10^3^ Hz).

The total oxide layer thickness can be estimated from the weight gains, assuming that the measured weight gain corresponds only to oxygen in zirconium oxide and that the oxide is stoichiometric ZrO_2_. In this case, the average layer thickness can be obtained from the relation

(2) $$\delta_{wg}=\frac{100\;\Delta G\;M_{ZrO_{2}}}{\rho_{ZrO_{2}}\;n\;M_{O}}=\frac{\Delta G}{0.0149}\;\left[\mu m\right]\;\;$$

where ∆*G* is the weight gain in g·dm^−2^, *M_ZrO2_* and *M_O_* are the molecular weight of the oxide and atomic weight of oxygen in g·mol^−1^, respectively, *n* is the oxygen stoichiometric coefficient (*n* = 2 in the case of ZrO_2_), and ρ ZrO_2_ is the zirconium oxide density = 5.71 g·cm^−3^. This relation was successfully applied in Zr corrosion studies [34,35] and gives reasonable results for zirconium oxide layers with thicknesses up to 30 μm. In our case, the results for the diamond-covered sample may have been slightly affected by the physical–chemical changes of the diamond layer during exposure to hot steam. Given the much higher weight of the oxide compared to diamond layer, the error should not be higher than units of %. Using relation (2), we obtained *δwg* = 24.8 μm and 22.1 μm for uncoated and diamond-coated samples, respectively. Knowing the approximate average oxide thickness enabled us to roughly assess the effective dielectric constant of the layer according to the relation

(3) $$\varepsilon_{r_{eff}}=\frac{\delta_{wg}C_{\infty }}{\varepsilon_{o}A}$$

where *δ_wg_* is the oxide thickness estimated from relation (9), *C_∝_* is the capacitance estimated from impedance data fitting, *ε_o_* is the vacuum dielectric constant (8.854 × 10^−14^ F·cm^−1^), and A is the area of the working electrode. The same relation was used to estimate the effective dielectric constant of the diamond coating. In this case, 500 nm was used as the coating thickness.

The results of the impedance spectra analysis and effective dielectric constant values are presented in Table 2.

The relatively high capacitance values at lower frequencies for samples not exposed to hot steam corresponded to the presence of a thin native oxide layer on the surface. The capacitance of the diamond layer coating was approximately 2.2 × 10^−8^ F·cm^−2^, which corresponded, for the 500 nm thick coating, to an effective relative dielectric constant of the coating of *ε_r_* ≈ 12. This value was higher than that found in a previous study (*ε_r_* = 6.7–9 for 300 nm PCD on Zircaloy 2, in [20]) and was relatively closer to that of graphite (*ε_r_* = 10–15) than to that of diamond (*ε_r_* = 5–6). This seems to be in conformity with the higher content of sp^2^-hybridized carbon found in the diamond coating. High resistances at low frequencies indicated insulating behavior (Figure 4). The behavior of the corrosion layer was, nevertheless, more complicated—the impedance response in the range of 10 kHz–1 MHz showed, at least in the cases of coated samples, another time constant, and so at least partly semi conductive behavior could be assumed.

The hot steam-exposed samples exhibited significant differences between the uncoated and coated samples. The capacitance of the uncoated sample was higher by a factor of 2, which implies a more than two times higher dielectric constant (*ε_r_* = 54) compared to the diamond-coated sample. It is worth noting that the expected values for zirconium oxide were approximately 22–23. The most likely explanation for such a high value might be the high level of microporosity and possible extensive penetration of water molecules into the structure of the oxide layer. In this case, the effective dielectric constant could be between that of zirconium oxide (22–23) and that of water (*ε_r_* = 80 at room temperature). The value of the oxide resistance seems to support this explanation, as it was more than 60× higher in the case of the diamond-coated sample, although the oxide thickness was 11% lower. Additionally, the impedance dispersion of the uncoated sample, which was already visibly higher from frequencies as high as 1000 Hz, seems to support this explanation. In contrast, a somewhat higher level of impedance dispersion appeared in the case of the diamond-coated sample in the MHz to kHz range. The reason is the response of the remnants of the diamond layer, probably having the form of dispersed carbon (sp^2^ and sp^3^) in the zirconium oxide matrix. The transformed carbon-containing layer was expected to have a much higher resistance than the original diamond coating (~1200 Ω·cm^2^ compared to 32 Ω·cm^2^). With a layer capacitance in the range of 8–9 × 10^−9^ F·cm^2^ and assuming a slightly lower value of the dielectric constant of such mixed dielectrics (*ε_r_* = 10–19) compared to zirconium oxide, the transformed carbon containing layer thickness could be 1–2 μm.

### 3.5. Ab Initio Calculations

DFT simulations were performed to investigate the effect of the diamond coating on the water dissociation at the oxide/water interface. We focused on carbon-containing complexes created on the ZIRLO surface and their impact on water dissociation probabilities. It has been published [21] that in the case of the diamond (containing sp^2^ carbon and >95% diamond)-coated ZIRLO, ZrC and ZrOC complexes are formed. Even if the diamond layer contains < 5% sp^2^ hybridized carbon, carbide formation (ZrC and ZrOC) occurs both in hot steam and hot water environments at the operating and accidental temperatures of the nuclear reactor [21].

Water dissociation and absorption energies were calculated on the ZrO_2_ (−111) surface with an oxygen vacancy and on the ZrO_2_ (−111) surface with an oxygen vacancy occupied by carbon (ZrOC). The absorption energy *E_abs_ ^H2O^* of the water molecule on the ZrO_2_ (−111) surface of the monoclinic phase was obtained:

(4) $$E_{abs}^{H_{2}0}=E_{surface+H_{2}0}-E_{surface}-E_{H_{2}0}$$

The water absorption energy was −0.31 eV for ZrO_2_ and slightly dropped to −0.32 eV for ZrO_2_ with oxygen vacancy (Table 3). However, this energy difference was negligible and could have been caused by the used level of simulations. The presence of C in the case of ZrOC lowered the absorption energy significantly to −0.77 eV. To investigate more possible inclusions of carbon, the water absorption energy on ZrC (111) surface was −0.55 eV, which is inferior to ZrO_2_ and ZrOV_O_ and superior to ZrOC. The described difference between ZrOC and ZrC was a result of the different bonding conditions of carbon. The negative sign of the absorption energy means that the absorption of the water molecule was more stable in comparison to the free state. This suggests that the water molecule was more robustly absorbed in cases of ZrOC and ZrC than in the cases without carbon atoms (ZrO_2_ and ZrOV_O_). Thus, the water molecule and ZrOC/ZrC created more stable complexes than water with ZrO_2_ or ZrOV_O_. Additionally, the zirconium carbide ZrC (111) surface was simulated to include another possible incorporation of carbon into the ZIRLO alloy.

Water dissociation was assumed to be the deprotonation of H_2_O. The thermodynamic control of the water dissociation should be dominant rather than the kinetic control due to the high temperature of the system. The reaction energies *E_r_* on the surfaces were calculated by DFT simulations as follows:

(5) $$E_{r}=E_{surface+OH^{-}}+E_{surface+H^{+}}-2E_{surface}-E_{H_{2}0}$$

Dissociation energies are presented in Table 4. The reaction energy for ZrOC was more than 1 eV lower than that for ZrO_2_. This difference was caused mainly by the stronger absorption of H+ on ZrOC than on ZrO_2_ or ZrOVo. For these cases, the water dissociation energies were comparable to that for ZrC. The investigation of the absorption energy of H+ (Table 4) indicated that the carbon atoms in ZrOC and ZrC were responsible for more robust interactions with H+. Therefore, due to the strong absorption of H+ on ZrOC or ZrC, the water dissociation equilibrium (cathodic/anodic processes) was disrupted. Subsequently, the water molecule dissociation was slower at the oxide/water interface, and the whole oxidation process was reduced. In contrast, the water dissociation on ZrC and ZrOC was more probable than water dissociation on ZrO_2_ molecules.

Secondarily, to investigate the influence of robust hydrogen absorption, water dissociation was modelled on the complex of ZrOC and H (ZrOC/H). The complex was considered to be electrically neutral due to the electron flux from Zr to the surface of ZrO_2_. The water dissociation energy on the surface with the ZrOC/H complex was significantly higher than the water molecule dissociation energy on the surface without hydrogen.

## 4. Discussion

### Corrosion of the Diamond-Coated ZIRLO

**Figure 6:**
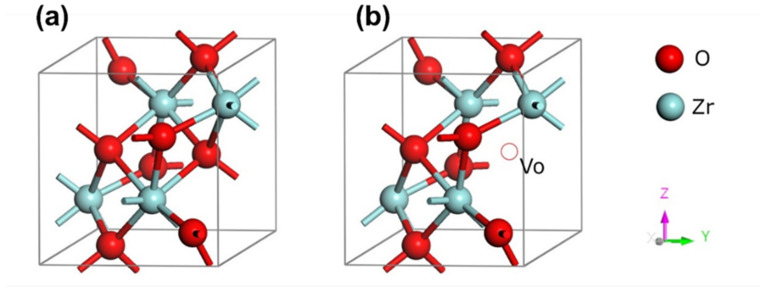
Monoclinic ZrO_2_ crystal structures (**a**) without and (**b**) with oxygen vacancy.

Diamond coating changes the conditions for ZIRLO hot steam/water corrosion by changing the physical (semi conductivity of ZrO_2_) properties and chemical (formation of ZrOC and ZrC groups) properties of ZIRLO, by which it affects the ZIRLO corrosion process. In a standard Zr oxidation/corrosion process, oxygen vacancies in ZrO_2_ (ZrOV_O_) (Figure 6) diffuse out from the oxide/metal interface towards the ZrO_2_ surface, and O^2^^−^ diffuses to the ZrO_2_/Zr interface [36,37]. When the oxygen anion reaches the metal/oxide interface [2,38,39], it reacts with zirconium, leading to the creation of ZrO_2_ [40] and the simultaneous release of four electrons [2,41]. These electrons migrate towards the oxide/steam interface to fill the empty levels of hydrogen ions—the reduction 4H^+^ + 4e^−^ → 2H_2_. Therefore, the growth of the oxide film by oxygen vacancy diffusion is the anodic process, and the reduction of hydrogen ions by electrons diffusing through the oxide film is the cathodic process (Supplementary Materials S2).

Zr oxide film grew on the Zr alloy surface with a large number of defects (oxygen vacancies—ZrOVo), which caused n-type semi conductivity, which means that electron donor defects in dielectric ZrO_2_ are localized in the forbidden gap close to the conductivity band [41], as seen in Figure 6.

**Figure 7:**
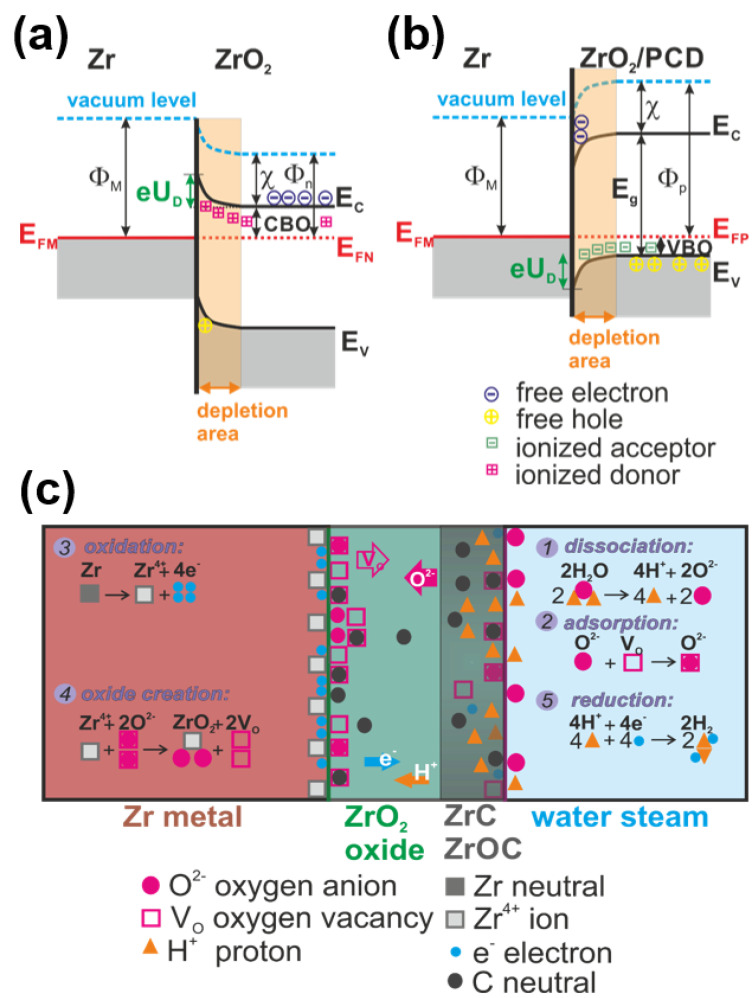
(**a**) Schottky barrier at the n-type semiconductor/metal interface. (**b**) Schottky barrier at the p-type semiconductor/metal interface. *U_D_* is barrier voltage, *Φ_M_* is the work function of the metal (Zr), *χ* is the semiconductor (ZrO_2_) electron affinity, and *Φn/Φp* is the semiconductor work function. (**c**) Scheme of the corrosion process in diamond coating on zirconium alloys. First, oxygen in the water molecule dissociates and is adsorbed onto the oxide layer surface at an oxygen vacancy site. Because of the defect concentration gradient and the electric potential across the oxide, the oxygen anions diffuse and react with zirconium cations to form new oxide. The formation of this new oxide releases electrons, which interact with the hydrogen ions at the cathodic site.

In our work [19], Mott–Schottky tests performed with ZIRLO samples pre-exposed to high temperature steam have shown plain n-type semi conductive behavior in the case of the non-coated samples, and mixed n- and p-type semi conductivity in the case of the PCD-coated samples. In the latter case, the corresponding donor and acceptor densities were 2–3 × 10^16^ cm^−3^ and 4.4 × 1,0^16^ cm^−3^, respectively. The Zr/ZrO_2_ behaved as a metal/n-type semiconductor junction making the space-charge area on the interface (Figure 7). For ZIRLO with an n-type (ZrOVo) semi conductive oxidized layer, O anions originating from the dissociation of water (2H_2_O → 2O^2^^−^ + 4H^+^) were attracted by the positive charge of the depletion layer at the n-semiconductor/Zr interface [2,41]. In addition, O anions had a lower probability to pass electron(s) through the n-type semi conductive ZrO_2_/Zr alloy interface. On the contrary the n-semiconductor/Zr interface enabled oxygen anions to be absorbed by oxygen vacancies at the ZrO_2_/Zr interface 2V_O_ + 2O^2−^ → 2O^2−^ and finally to interact with the zirconium Zr^4+^ + 2O^2−^ → ZrO_2_ + 2V_O_.

In [19,21,24], we presented that the diamond-coated Zr nuclear fuel rods were protected against accidental temperature hot steam/water corrosion due to the fact that the carbon diffused from the diamond coating into ZrO_2_, changing its physical property of semi conductivity. ZrO_2_ under diamond coating behaved as a compensated semiconductor with mixed n- and p-type characteristics (Figure 7). In the case of n/p semi conductivity of ZrO_2_, the oxygen anion from the dissociation of water was not so strongly attracted to the Zr/ZrO_2_ interface area, and when it reached the Zr/ZrO_2_ interface, with higher probability it lost electrons, reducing the probability to interact with zirconium vacancies. Such effects disrupted the oxidation process on the anodic side of the corrosion loop (Figure 7). This protection is stronger with growing temperature and/or time when carbons even from diamond nanocrystals are released and penetrate into the deeper parts of ZrO_2_ [21]. We calculated that by adding carbon from the diamond coating into ZrO_2_, the electric field generated by space charge at the Zr/ZrO_2_ interface was reduced from 1.445 × 10^−7^ V to 1.083 × 10^−7^ V (Supplementary Materials S3, Table S1).

As far as chemical and electrochemical changes caused by diamond coating are concerned, we showed that ZrOC and ZrC groups were formed on the ZIRLO surface. ZrOC and ZrC groups attract hydrogen-creating H^+^ and ZrOC, and H^+^ and ZrC complexes. Due to the strong absorption of H^+^ on ZrOC and ZrC, the water dissociation equilibrium (cathodic and anodic balance) is disrupted, reducing the oxidation process. Furthermore, ZrOC and ZrC groups contribute to the limited incorporation of hydrogen into the ZIRLO rod surface. The uptake of hydrogen into uncoated ZIRLO was an order of magnitude higher than hydrogen uptake into carbon-coated ZIRLO samples after all tested hot steam treatments.

According to our accidental temperature tests, corrosion of the coated ZIRLO began to be affected by carbon diffusion (changing chemical and physical ZrO_2_ properties) approximately after 2.5 min in 900 °C steam. After 20 min at 900 °C in a hot steam furnace, the oxidation of the diamond coated ZIRLO rod was strongly affected by carbon diffusion; coated ZIRLO corrosion was reduced by ~20% compared to that of the uncoated ZIRLO rod treated under the same conditions. The coating had also a stabilizing effect on the tetragonal ZrO_2_ phase and reduced the number of cracks in the ZrO_2_ layer.

However, when the steam temperature was raised above 1000 °C (30 min in a 900 °C steam furnace and 20 min in a 1000 °C steam furnace), the oxidation of the diamond layer-coated ZIRLO rods was reduced only by 7% compared to that of the uncoated ZIRLO rods. At such a high temperature as 1000 °C, the complexes H+/ZrOC and H+/ZrC could be unstable. The loss of hydrogen from H+/ZrOC or H+/ZrC complexes after the 1000 °C hot steam treatment (compared to 900 °C treatment) was confirmed by Raman spectroscopy. Water dissociation on ZrC and ZrOC is more probable compared to water dissociation on H+/ZrOC or H+/ZrC and even on ZrO_2_ molecules. Very dynamic changes in the formation and disintegration of all the complexes and the carbon diffusion rate affect conditions for accidental temperature water dissociation. These changes, which strongly depend on the temperature and diamond content in the coating, support and also reduce corrosion of ZIRLO fuel rods. According to our previous accidental temperature tests of polycrystalline diamond coated nuclear rods [19] in 1000 °C hot steam/water (60 min) and in 1100 °C (60 min) and 1200 °C (20 min) hot steam/water, the corrosion of diamond layer coated nuclear tubes was always lower than the corrosion of uncoated rods subjected to the same treatment [19].

The ability of the diamond coating to protect ZIRLO against hot steam corrosion depends on the fraction of the sp^2^ carbon phase in the coating. Soft, sp^2^ carbon controls the amount of the ZrOC/ZrC complexes and thus the electrochemical balance. If more soft carbon is available to make more H+/ZrOC and H+/ZrC complexes than at temperatures around 1000 °C, the hydrogen ions are released from complexes, and conditions for water dissociation are improved. When the ZIRLO nuclear fuel rods surface was coated by a PCD layer containing >95% of the diamond phase, the corrosion was after 60 min in 1000 °C hot steam reduced by 25% [19,20].

## 5. Conclusions

In this work we described and explained the influence of diamond coating (60% diamond) on chemical and physical processes and states that affect the ZIRLO corrosion at nuclear reactor accidental temperatures:

- Carbon from diamond coating changes ZrO_2_ layer semi conductivity from the n-type to the n/p mixture type. Due to such material modification, the electric field at yhe Zr/ZrO_2_ interface is reduced and limits the Zr oxidation. After 20 min at 900 °C in a hot steam furnace, the oxidation of the diamond-coated ZIRLO rod was strongly affected by carbon diffusion: coated ZIRLO corrosion was reduced by ~20% compared to that of the uncoated ZIRLO rod treated under the same conditions. This protection is stronger with growing temperature and time when carbon atoms even from diamond nanocrystals are released and penetrate into the deeper parts of ZrO_2_.
- “Soft” carbon from diamond coatings supports establishment of ZrC and ZrOC complexes, which interact with H+. ZrC/H+ and ZrOC/H+ complexes disturb the water dissociation equilibrium (cathodic and anodic balance). However, the diamond coating protection declined at 1000 °C (oxidation of the diamond layer-coated ZIRLO rods was reduced just by 7% compared to that of the uncoated ZIRLO rods), and we suppose that this effect is strongly influenced by the complexes H+/ZrOC and H+/ZrC high temperature instability. At steam temperatures around 1000 °C, the H+/ZrOC and H+/ZrC complexes are massively destroyed, and better conditions for water dissociation on ZrOC and ZrC groups cause an increase of the ZIRLO corrosion.

## Figures and Tables

**Table 1:** Hot steam treated ZIRLO oxidation. Values of the weight gain (given in % and mg dm^−2^) for ZIRLO protected by diamond coating and uncoated ZIRLO rods after the exposure to the hot steam treatment. Note: the recalculated (normalized) values of weight gains of the coated parts of the samples are given.

ZIRLO Rod Sample	Relative Weight Gain (% of ZIRLO wg) 850 °C	Weight Gain (mg∙dm^−2^) 850 °C	Relative Weight Gain (% of ZIRLO wg) 900–1000 °C	Weight Gain (mg∙dm^−2^) 900–1000 °C
Coated	84	700	93	1134
Bare	100	790	100	1220

**Table 2:** Impedance parameters estimated by approximating experimental data with the impedance of the equivalent circuits in Figure 5; diamond coating and oxide thicknesses and effective dielectric constants as estimated from relation (3).

Scheme	Remark	*R_diam_*	*C_diam_*	*D_diam_*	*ε_diam_*	*R_ox_*	*C_ox_*	*d_ox_*	*ε_OX_*
		(Ω·cm^2^)	(F·cm^−2^)	(µm)		(Ω·cm^2^)	(F·cm^−2^)	(µm)	
ZIRLO	bare					4 × 10^7^	1.7 × 10^−6^		
ZIRLO + diam	0.5 μm diam	32	2.2 × 10^−8^	0.5	12	9 × 10^7^	2.2 × 10^−7^		
ZIRLO	40 min, 850 °C					1.2 × 10^9^	1.8 × 10^−9^	25	54
ZIRLO + diam	40 min, 850 °C	1188	8.5 × 10^−9^	1–2	10–19	7.4 × 10^10^	9.5 × 10^−10^	22	21

**Table 3:** Values of absorption energy of water molecule on the (−111) surface of ZrO_2_, ZrO_2_ (−111) surface with an oxygen vacancy (ZrOV_O_), ZrO2 (−111) surface with oxygen vacancy occupied by carbon (ZrOC), and the ZrC (111) surface.

Surface Type	$E_{abs}^{H_{2}O}$ (eV)
ZrO_2_	−0.31
ZrOV_O_	−0.32
ZrOC	−0.77
ZrC	−0.55

**Table 4:** Dissociation energy *E_r_* of the water molecule and absorption energy *E_abs_ ^H^*^+^ of H+ on the ZrO_2_ (−111) surface, ZrO_2_ (−111) surface with an oxygen vacancy (ZrOVo), ZrO2 (−111) surface with an oxygen vacancy occupied by C (ZrOC), and the ZrC (111) surface.

Surface Type	$E_{r}$ (eV)	$E_{abs}^{H^{+}}$ (eV)
ZrO_2_	−0.51	−17.12
ZrOVo	−0.63	−16.87
ZrOC	−1.59	−18.14
ZrC	−0.59	−19.57

## Data Availability

The data are available and will be provided on request.

## Supplementary Materials

The following are available online at https://www.mdpi.com/article/10.3390/ma14216315/s1, Figure S1: Raman spectra of carbon coating containing less than 60% of diamond phase measured across ZIRLO tube circumference. The spectra were taken with the step of 45° around the circumference and at each step three spectra were measured. Note: the peak indicated by an asterisk is assigned to CaCO3, Figure S2: Raman spectrum of the ZIRLO tube coated with carbon coating after 850 °C (blue curve); spectrum after the hot steam treatment at 900–1000 °C (black curve), Figure S3: Monoclinic ZrO2 (−111) surface with 10Å in vacuum. Table S1: Quantities used for calculation of the voltage.
