# Supplementary material for: Diamond Coating Reduces Nuclear Fuel Rod Corrosion at Accidental Temperatures: The Role of Surface Electrochemistry and Semiconductivity
Source: Materials (Basel). 2021 Oct 22;14(21):6315. doi: 10.3390/ma14216315 (PMC8585298; doi:10.3390/ma14216315)
Supplement: Supplementary file 1 [file materials-14-06315-s001.zip › materials-1396175-supplementary.pdf]

materials

Supplementary Materials

Diamond Coating Reduces Nuclear Fuel Rod Corrosion at Accidental Temperatures: The Role of Surface Electrochemistry and Semiconductivity

Lucie Celbová [1,2], Petr Ashcheulov [1], Ladislav Klimša [1], Jaromír Kopeček [1], Kateřina Aubrechtová Dragounová [1,2], Jakub Luštinec [1,2], Jan Macák [3], Radek Škoda [4] and Irena Kratochvílová [1,2,*]

1 Institute of Physics of the Czech Academy of Sciences, Na Slovance 1999/2, 182 21 Prague, Czech Republic; celbova@fzu.cz (L.C.); ashcheulov@fzu.cz (P.A.); klimsa@fzu.cz (L.K.); kopecek@fzu.cz (J.K.); dragounova@fzu.cz (K.A.D.); lustinec@fzu.cz (J.L.)
2 Faculty of Nuclear Sciences and Physical Engineering, Czech Technical University in Prague, Břehová 7, 115 19 Prague, Czech Republic
3 Power Engineering Department, University of Chemistry and Technology, Technická 3, 166 28 Prague, Czech Republic; Jan.Macak@vscht.cz
4 Czech Institute of Informatics, Robotics and Cybernetics, Czech Technical University in Prague, Jugoslávských partyzánů 1580/3, 160 00 Prague, Czech Republic; Radek.Skoda@cvut.cz
* Correspondence: krat@fzu.cz; Tel.: +420-723-814-810

Contents:

S1. Raman spectroscopy
S2. ZrO_2 surface layer and defects in ZrO_2
S3. Zr/ZrO_2 interface : Schottky barrier

References

S1. Raman spectroscopy

As discussed in our previous works, thin coating of polycrystalline diamond (PCD) fabricated via chemical vapor deposition (CVD) techniques presents an effective barrier-type protection against high-temperature induced corrosion of Zr-alloy (ZIRLO) tube rods, primarily due to the release of carbon atoms from the coating and their consequent diffusion into the underlying ZIRLO material [1-3]. Effectively, availability of carbon atoms accessible for a prompt penetration/diffusion into the ZIRLO surface defines the degree of favourable modification of the properties of the Zr material. Therefore, Raman measurements were taken across the ZIRLO tube circumference, to access the homogeneity of the as deposited carbon coating along with the coating composition (sp^3 and sp^2 phases). Each of the spectra exhibited a signal from the carbon coating even though a prevalence of them very weak (**Fig. S1**). The type of the spectrum from the uppermost left inset in **Fig. S1** corresponds to the spectrum in **Fig. 2b** (see main text).

Raman spectra of ZIRLO tube with carbon coating after the exposure to 850°C and 900-1000°C hot steam treatment are homogeneous throughout the tube. The typical Raman spectra are depicted on **Fig. S2**.

Raman measurements revealed that the carbon layer had graphitized and the diamond peak is not visible in the spectra after the hot steam treatment. The m/I_G (m is the slope of the linear photoluminescence background (not shown), I_G is the intensity of the G band) parameter which is used for estimation of hydrogen content from Raman spectra is ~5-6 times higher for the tube after 850°C water treatment than for the tube after 900-1000°C water treatment which can be possibly correlated with peaks at ~1400-1500 cm^{-1} which also exhibit some degree of internal structure after the 850 °C treatment and we thus assign those to the presence of polycyclic aromatic hydrocarbon (PAH) units (blue curve in **Fig. S2**). After 900-1000°C water treatment, the m/I_G ratio significantly decreases together with the intensity of those peaks suggesting that PAH units lost some of their hydrogens and graphitized. Both of the spectra from **Fig. S2** exhibit D peak at ~1360 cm^{-1}, G peak at 1600 cm^{-1}, D′ peak at ~1620 cm^{-1} and the 850 °C-treated tube further exhibits -OH groups peak at ~1650 cm^{-1} [4].

Overall, Raman spectra analysis suggests that after 850°C water treatment, PCD layer on ZIRLO tube transformed to nano-graphitic disordered domains and PAH units which subsequently disappear after 900-1000°C steam treatment, thus resulting in the ZIRLO tube covered by nano-graphitic disordered domains embedded in the amorphous sp^2 carbon.

S2. ZrO_2 surface layer and defects in ZrO_2

A sub-band under the edge of conduction ZrO_2 band is formed predominantly by d-states of Zr. The defect sub-band width depends on vacancies concentration and with the reduction of the concentration it degenerates into a vacancy level. Zr electron configuration ($1s^2$ $2s^2p^6$ $3s^2p^6d^{10}$ $4s^2p^6d^2$ $5s^2$), and for for the Zr atom the $4s^2$, $4p^6$, $4d^2$, $5s^2$ electrons are explicitly treated as valence electrons. For O atom, the configuration is $2s^2$ $2p^4$, where $2s^2$ and $2p^4$ electrons are explicitly treated as valence electrons. The O 2p states make the top part of the valence band of ZrO_2 formed by a small admixture of metal d states. The conduction bands are formed by d-states of metals. That means that valence bands in ZrO_2 are formed mostly by oxygen states, while conduction band is formed by Zr 4d states. In ZrC, the mixture of C 2p and Zr 4d and 4p orbitals around the Fermi level makes a large contribution to the covalent bonds between the Zr and C atoms in the ZrC matrix. The Bader analysis shows that the valency of ZrC can be expressed as $Zr^{+1.71}C^{-1.71}$.

The gaps between the sub-band and conduction band are 0.44 eV for ZrO2, with the width of 0.51 eV, and the bandgap is 3.40 eV. When an oxygen atom is removed from ZrO_2 the remaining two electrons are forced to reside in Zr 4d orbitals (in conduction band) – the oxygen vacancies are n-type defects in ZrO_2. When an oxygen atom in ZrO_2 is replaced by carbon with electron configuration $2s^2$ $2p^2$ two valence electrons in 2p orbitals are missing, i.e. hole conduction in ZrO_2 valence band can be obtained during the high temperature corrosion process (Zr-O-C), therefore changing the electrical properties of ZrO_2.

Overall, the growth of oxide film happens as a result of diffusion of anion vacancy through the oxide layer. Generation of vacancies happens on oxide/metal interface. Oxygen atoms can "jump over" into interstitial states of contacting metal forming oxygen vacancies in the oxide due to thermal fluctuations. The rate of this reaction is supposed to depend only on temperature. Oxygen ions move towards metal by anion vacancies according to gradient of vacancies in the oxide film. With presence of oxidizing medium on the outside surface of the oxide film, the film composition is close to stoichiometric. Deviation from stoichiometry is considerable on the boundary with metal. If there are water molecules near the surface of the oxide film then a negatively charged oxygen ion and two positively charged hydrogen ions are formed on the surface as a result of dissociation. These two protons can be neutralized by electrons with formation of hydrogen atoms.

S3. Zr/ZrO_2 interface : Schottky barrier

The Zr/ZrO_2 interface under ZrO_2 part of ZIRLO fuel rod behaves as metal/n-type semiconductor Schottky junction, which results in the presence of space-charge area on the interface. This area without mobile charges, called depletion region, should be seen as charged capacitor. The voltage corresponding to the Schottky barrier can then be expressed via the equality between the work performed in charge/discharge and the barrier height:

$$W = \frac{1}{2} Q_{SC} U_D = \Phi_M - \chi - CBO = \Phi_M - \Phi_n, \quad (1)$$

where Φ_M is the work function of the metal, χ is the electron affinity and *CBO* is the conduction band offset of the n-type semiconductor, which can be combined into the work function Φ_n.

The charge in the depletion region is written as

$$Q_{SC} = A\sqrt{2e\varepsilon_0\varepsilon_r N_D \left(U_D - \frac{kT}{e}\right)}, \quad (2)$$

where A is the area of the depletion region, N_D is the concentration of donors in the depletion region and ε_r is the effective relative dielectric permittivity of ZrO_2, taken from [3]. Finally, for the barrier voltage,

$$U_D^{n-type} = \sqrt[3]{\frac{2(\Phi_M - \Phi_n)^2}{A^2 e\varepsilon_0\varepsilon_r N_D}}. \quad (3)$$

Values of given quantities were partly obtained from references and partly inferred from previous measurements, reported in [3,5,6]. We assumed an area A of 1 μm² for the depletion region. A summary is given in **Table S1**.

For calculation of the Schottky barrier voltage for the Zr/ZrO_2/diamond coating, partial compensation of donor level N_D by acceptor level N_A is expected from the result of [3]. We assumed the same area A,

$$Q_{SC} = A\sqrt{2e\varepsilon_0\varepsilon_{reff}(N_D - N_A)\left(U_D - \frac{kT}{e}\right)}, \quad (4)$$

where the quantities have similar meanings as the n-type semiconductor quantities, and ε_{reff} is the effective dielectric constant for our semiconductor system, reported in [5] [3] and summarized in **Table S1**. We used the relations between the electron affinity of a p-type semiconductor and the Zr work function and defined the work function for a p-type semiconductor as

$$eU_D = -\phi_M + \chi + E_g - VBO = -\phi_M + \phi_p, \quad (5)$$

where E_g is the ZrO_2 gap and *VBO* is the valence band offset. Then, the expression for voltage should be

$$U_D^{p-type} = \sqrt[3]{\frac{2(-\Phi_M+\Phi_p)^2}{A^2 e\varepsilon_0\varepsilon_{reff}(N_D-N_A)}}. \quad (6)$$

Table S1. Quantities used for calculation of the voltage. .

Parameter	***Zr***	***Zr/ZrO₂***	***Zr/ZrO₂/carboncoating***
Work function [eV]	4.1 [5]	2.0 [3]	4.0 (deduced from [3])
N_D [cm^{-3}]	-	2.3 × 10^{18} [3]	2-3 × 10^{16} [3]
N_A [cm^{-3}]		-	4.4 × 10^{16} [3]
Effective dielectric constant $\varepsilon_r/\varepsilon_{reff}$ [-]		23 [3]	10 – 17 [3]
Band gap ZrO_2 E_g [eV]		5.7 [5]	

References

1. Kratochvilova, I.; Skoda, R.; Skarohlid, J.; Ashcheulov, P.; Jager, A.; Racek, J.; Taylor, A.; Shao, L. Nanosized polycrystalline diamond cladding for surface protection of zirconium nuclear fuel tubes. *Journal of Materials Processing Technology* **2014**, *214*, 2600-2605, doi:10.1016/j.jmatprotec.2014.05.009.
2. Ashcheulov, P.; Skoda, R.; Skarohlid, J.; Taylor, A.; Fekete, L.; Fendrych, F.; Vega, R.; Shao, L.; Kalvoda, L.; Vratislav, S., et al. Thin polycrystalline diamond films protecting zirconium alloys surfaces: From technology to layer analysis and application in nuclear facilities. *Applied Surface Science* **2015**, *359*, 621-628, doi:10.1016/j.apsusc.2015.10.117.
3. Skarohlid, J.; Ashcheulov, P.; Skoda, R.; Taylor, A.; Ctvrtlik, R.; Tomastik, J.; Fendrych, F.; Kopecek, J.; Chab, V.; Cichon, S., et al. Nanocrystalline diamond protects Zr cladding surface against oxygen and hydrogen uptake: Nuclear fuel durability enhancement. *Scientific Reports* **2017**, *7*, 14, doi:10.1038/s41598-017-06923-4.
4. Mochalin, V.N.; Shenderova, O.; Ho, D.; Gogotsi, Y. The properties and applications of nanodiamonds. *Nat. Nanotechnol.* **2012**, *7*, 11-23, doi:10.1038/nnano.2011.209.
5. Fulton, C.C.; Lucovsky, G.; Nemanich, R.J. Electronic properties of the Zr-ZrO2-SiO2-Si(100) gate stack structure. *J. Appl. Phys.* **2006**, *99*, 10, doi:10.1063/1.2181282.
6. Kratochvilova, I.; Ashcheulov, P.; Skarohlid, J.; Skoda, R.; Kopecek, J.; Sajdl, P.; Macak, J.; Lajcinova, M.; Novakova, A.; Neethling, J., et al. Zr alloy protection against high-temperature oxidation: Coating by a double-layered structure with active and passive functional properties. *Corrosion Sci.* **2020**, *163*, 11, doi:10.1016/j.corsci.2019.108270.

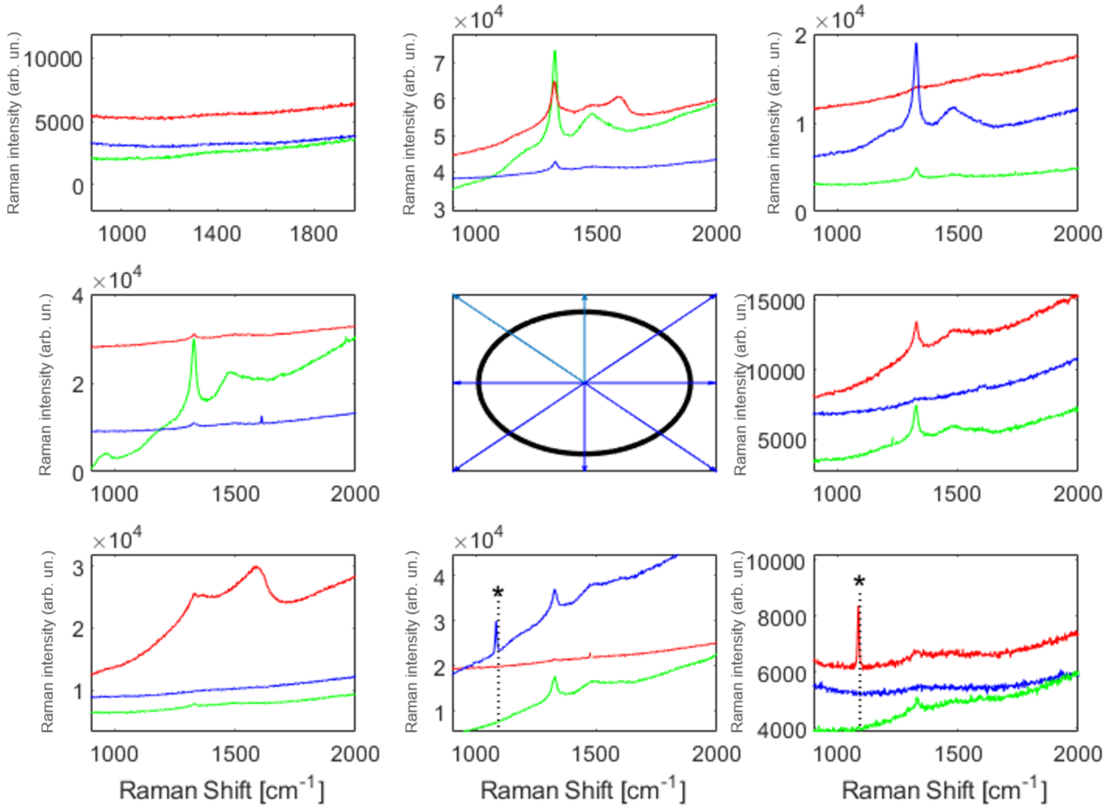

Figure S1. Raman spectra of carbon coating containing less than 60% of diamond phase measured across ZIRLO tube circumference. The spectra were taken with the step of 45° around the circumference and at each step three spectra were measured. Note: the peak indicated by an asterisk is assigned to $CaCO_3$.

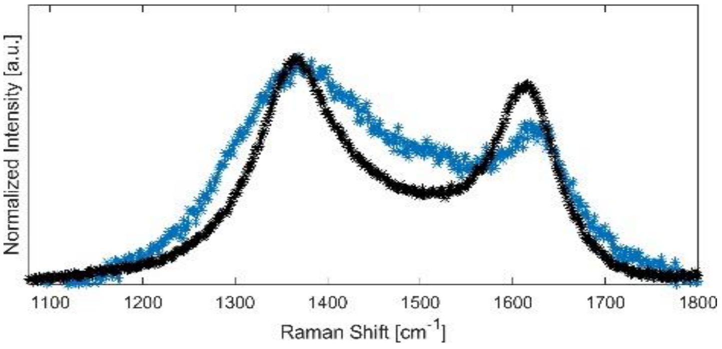

Figure S2. Raman spectrum of the ZIRLO tube coated with carbon coating after 850°C (blue curve); spectrum after the hot steam treatment at 900-1000°C (black curve).

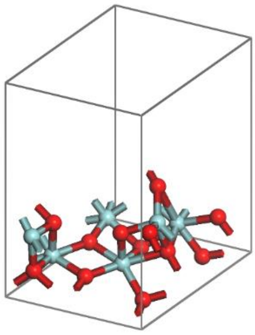

Figure S3. Monoclinic ZrO_2 (-111) surface with 10Å in vacuum.
